# Exploring Informal Caregivers’ Priorities of Nursing Home Dementia Care From Communities of Color

**DOI:** 10.1093/geroni/igab046.2101

**Published:** 2021-12-17

**Authors:** Stephanie Rouch, Marybeth Moscirella, Alexandra Harper, Cara Lekovitch, Rose Turner, Catherine Piersol, Natalie Leland

**Affiliations:** 1 University of Pittsburgh, Pittsburgh, Pennsylvania, United States; 2 University of Pittburgh, Pittsburgh, Pennsylvania, United States; 3 Thomas Jefferson University, Philadelphia, Pennsylvania, United States

## Abstract

Informal caregivers are critical stakeholders in nursing home (NH) care for individuals with dementia. Given racial and ethnic disparities in United States NHs, there is a need to understand informal caregivers’ perspectives, particularly among those that identify as members of a community of color. We conducted a scoping review of informal caregiver priorities of nursing home dementia care. Included studies exclusively examined priorities of informal caregivers identifying as Black, Indigenous, or people of color. The final sample (n=12) included two United States studies representing African American and Korean informal caregivers. The remaining studies were conducted in other countries. Informal caregivers expressed a desire for professional support during the nursing home transition, increased staff knowledge of dementia, and improved resident engagement. These findings highlight the paucity of informal caregivers identifying as Black, Indigenous, or people of color represented in US nursing home dementia research. Future efforts must include communities of color.

